# Age‐specific, population‐level pedigree of wild black bears provides insights into reproduction, paternity, and maternal effects on offspring apparent survival

**DOI:** 10.1002/ece3.8770

**Published:** 2022-03-31

**Authors:** Melissa J. Reynolds‐Hogland, Alan B. Ramsey, Carly Muench, Kristine L. Pilgrim, Cory Engkjer, Philip W. Ramsey

**Affiliations:** ^1^ Wildlife Research and Education Foundation Frenchtown Montana USA; ^2^ MPG Ranch Florence Montana USA; ^3^ USDA National Genomics Center Rocky Mountain Research Station Missoula Montana USA

**Keywords:** black bear, genetic data, maternal effects, pedigree, reproduction, video data

## Abstract

Wildlife pedigrees provide insights into ecological and evolutionary processes. DNA obtained from noninvasively collected hair is often used to determine individual identities for pedigrees and other genetic analyses. However, detection rates associated with some noninvasive DNA studies can be relatively low, and genetic data do not provide information on individual birth year. Supplementing hair DNA stations with video cameras should increase the individual detection rate, assuming accurate identification of individuals via video data. Video data can also provide birth year information for individuals captured as young of the year, which can enrich population‐level pedigrees. We placed video cameras at hair stations and combined genetic and video data to reconstruct an age‐specific, population‐level pedigree of wild black bears during 2010–2020. Combining individual birth year with mother–offspring relatedness, we also estimated litter size, interlitter interval, primiparity, and fecundity. We used the Cormack‐Jolly‐Seber model in Program Mark to evaluate the effect of maternal identity on offspring apparent survival. We compared model rankings of apparent survival and parameter estimates based on combined genetic and video data with those based on only genetic data. We observed 42 mother–offspring relationships. Of these, 21 (50%) would not have been detected had we used hair DNA alone. Moreover, video data allowed for the cub and yearling age classes to be determined. Mean annual fecundity was 0.42 (95% CI: 0.27, 0.56). Maternal identity influenced offspring apparent survival, where offspring of one mother experienced significantly lower apparent survival (0.39; SE = 0.15) than that of offspring of four other mothers (0.89–1.00; SE = 0.00–0.06). We video‐documented cub abandonment by the mother whose offspring experienced low apparent survival, indicating individual behaviors (e.g., maternal care) may scale up to affect population‐level parameters (e.g., cub survival). Our findings provide insights into evolutionary processes and are broadly relevant to wildlife ecology and conservation.

## Graphical abstract

We installed video cameras at hair stations and combined genetic and video data to reconstruct an age‐specific, population‐level pedigree of wild black bears during 2010–2020. Combining individual birth year with mother‐offpsring relatedness, we also estimated reproductive parameters and evaluated the effect of maternal identity on offspring survival. Maternal identity influenced offspring survival, where offspring of one mother experienced significantly lower apparent survival (0.39; SE = 0.15) than that of offspring of four other mothers (0.89–1.00; SE = 0.00–0.06)

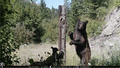

## 1 INTRODUCTION

Estimating relatedness among individuals of wildlife populations is often key to fully understanding ecological and evolutionary processes (Firth et al., 2015; Kruuk & Hill, 2008; Widdig et al., 2001), including inbreeding effects (Kardos et al., 2018; Liberg et al., 2005; Xue et al., 2015), kinship (Patel et al., 2017; Reid et al., 2016), gene flow and source‐sink dynamics (Draheim et al., 2016; Figueiredo‐Vázquez et al., 2021), genetic diversity (Paetkau et al., 1998; Wultsch et al., 2016), and immigration (Morandin et al., 2014; Wooding & Ward, 1998). Increasingly, relatedness data are used to reconstruct population‐level wildlife pedigrees (Eggert et al., 2010; Liberg et al., 2005; Richards‐Zawacki et al., 2012), which are a crucial part of individual‐based projects (Pemberton, 2008) and can be important to conservation planning and management (Khan et al., 2020). For example, Giglio et al. (2018) reconstructed a population‐level pedigree for bison (*Bison bison*) to evaluate multiple management strategies aimed at retaining genetic variation.

To reconstruct wildlife pedigrees and evaluate other processes using genetic data, blood or muscle tissue collected from captured animals is often used (Clutton‐Brock & Pemberton, 2004; Firth et al., 2015; Liberg et al., 2005). However, this method can be challenging to implement for long‐lived, elusive species such as large carnivores (Beier et al., 2005; Khan et al., 2020). An alternative approach is to use hair DNA collected from noninvasive hair stations, which has been successfully used to estimate large carnivore abundance (Immell & Anthony, 2008; López‐Bao et al., 2018), density (Hooker et al., 2015; Loosen et al., 2018; Stenglein et al., 2010), and apparent survival (Boulanger et al., 2004; Pederson et al., 2012). Even a population‐level pedigree of grizzly bears (*Ursus arctos*) was reconstructed using primarily hair DNA (Kendall et al., 2016). Despite these successes, using hair DNA alone to estimate population‐level processes can be limiting when the probability of genetic capture is low. For example, Gurney et al. (2020) placed trail cameras at American black bear (*Ursus americanus*) hair stations and found that 32% of bear visits resulted in a bear approaching a station, but not entering or leaving hair.

Understanding this shortcoming can be leveraged to improve genetic‐based studies that use hair DNA data. For example, we previously demonstrated that video data from cameras installed at hair stations increased the probability of detecting individual black bears (Reynolds‐Hogland et al., *2022*). We could video‐capture individuals that visited hair stations but did not deposit hair, provided individuals were close enough to cameras to trigger them. This method assumes individuals can be accurately identified via video data. For wild black bears, we successfully identified individuals through meticulous scrutiny of video data (not still photos) cross‐referenced with individual genetic identities determined from hair DNA concurrently collected at hair stations in view of video cameras (Ramsey et al., 2019; Reynolds‐Hogland et al., *2022*). Advances in automated facial recognition software (Clapham et al., 2020; Schneider et al., 2020) should make it more feasible to identify individuals using remote photography, without the need for meticulous scrutiny of video data. Thus, incorporating video cameras at hair DNA stations promises increased individual detection rates, which should help improve the accuracy of parameter estimation (e.g., occupancy, abundance, density, etc.).

In addition to low detection probabilities associated with some hair DNA studies, genetic data currently do not provide information on individual age. Such information would enrich population‐level pedigrees with the incorporation of individual birth year. Integrating remote photography at hair stations can provide a noninvasive means for determining individual birth year for those animals captured as young of the year. For example, first‐year black bears captured via video are easily identified as cubs.

When population‐level wildlife pedigrees include information on both individual birth year and mother–offspring relatedness, reproductive parameters can also be estimated. For black bears, reproductive parameters such as fecundity, natality, litter size, interlitter interval, and primiparity have been estimated using live‐capture data (Baldwin & Bender, 2009; Beecham, 1980; Costello et al., 2003; Garrison et al., 2007; Hebblewhite et al., 2003; Kasworm & Thier, 1994). In addition, Aune and Anderson (2003) used DNA from harvested bears to estimate interlitter interval, Garrison et al. (2007) used data from den visits and remote photography to determine litter size and cub survival, and Miller (1994) estimated litter size based on data collected during den visits and observations of litters after den emergence. Several bear studies have used hair DNA and the Pradel model (Pradel, 1996) to estimate recruitment rate or rate of addition (*f*; Boulanger et al., 2002; Boulanger et al., 2004; Pederson et al., 2012; Sawaya et al., 2012; McCall et al., 2013). To date, no study has estimated natality, fecundity, litter size, interlitter interval, or primiparity using only video and genetic data.

Assuming young offspring can be tracked through time via genetic and video data, offspring apparent survival can also be estimated using noninvasive data. When maternity is also known, it should be possible to evaluate maternal effects on offspring apparent survival. A maternal effect occurs when an offspring's phenotype is shaped by the properties of the mother, independent of the mother's genotype (Bernardo, 1996), although maternal properties may have a genetic basis (Ramakers et al., 2018). Both the environment and behavior may shape maternal effects as variation in maternal environment (e.g., availability of food resources) or maternal behavior (e.g., maternal care of offspring) results in variation in offspring phenotype (Mousseau & Fox, 1998). For example, maternal effects strongly influence offspring mass gain (Skibiel et al., 2009), which positively correlates with offspring survival (Clutton‐Brock et al., 1982; Côté & Festa‐Bianchet, 2001; Dahle et al., 2006). For bears, the effects of maternal body mass (Derocher & Stirling, 1998; Rode et al., 2020; Samson & Huot, 1995; Stringham, 1990) and maternal age or experience (Elowe & Dodge, 1989; Garrison et al., 2007; Johnson et al., 2020; Zedrosser et al., 2009) on offspring mass and offspring survival have been evaluated. Also, Zedrosser et al. (2013) assessed the effect of maternal identity on grizzly bear yearling survival. Currently, it is unknown how maternal identity may affect the survival of black bear offspring.

We used genetic data supplemented with video data to evaluate population‐level processes of wild black bears living on a conservation property in western Montana, USA. Our research objectives were to (1) reconstruct a population‐level pedigree with individual birth years, (2) estimate natality, litter size, interlitter interval, primiparity, and fecundity using genetic data integrated with video data, and, (3) evaluate the effects of maternal identity on offspring apparent survival using combined genetic and video data and compare the resulting model rankings and parameter estimates with those based on only genetic data.

## 2 METHODS

### 2.1 Study site

**FIGURE 1:**
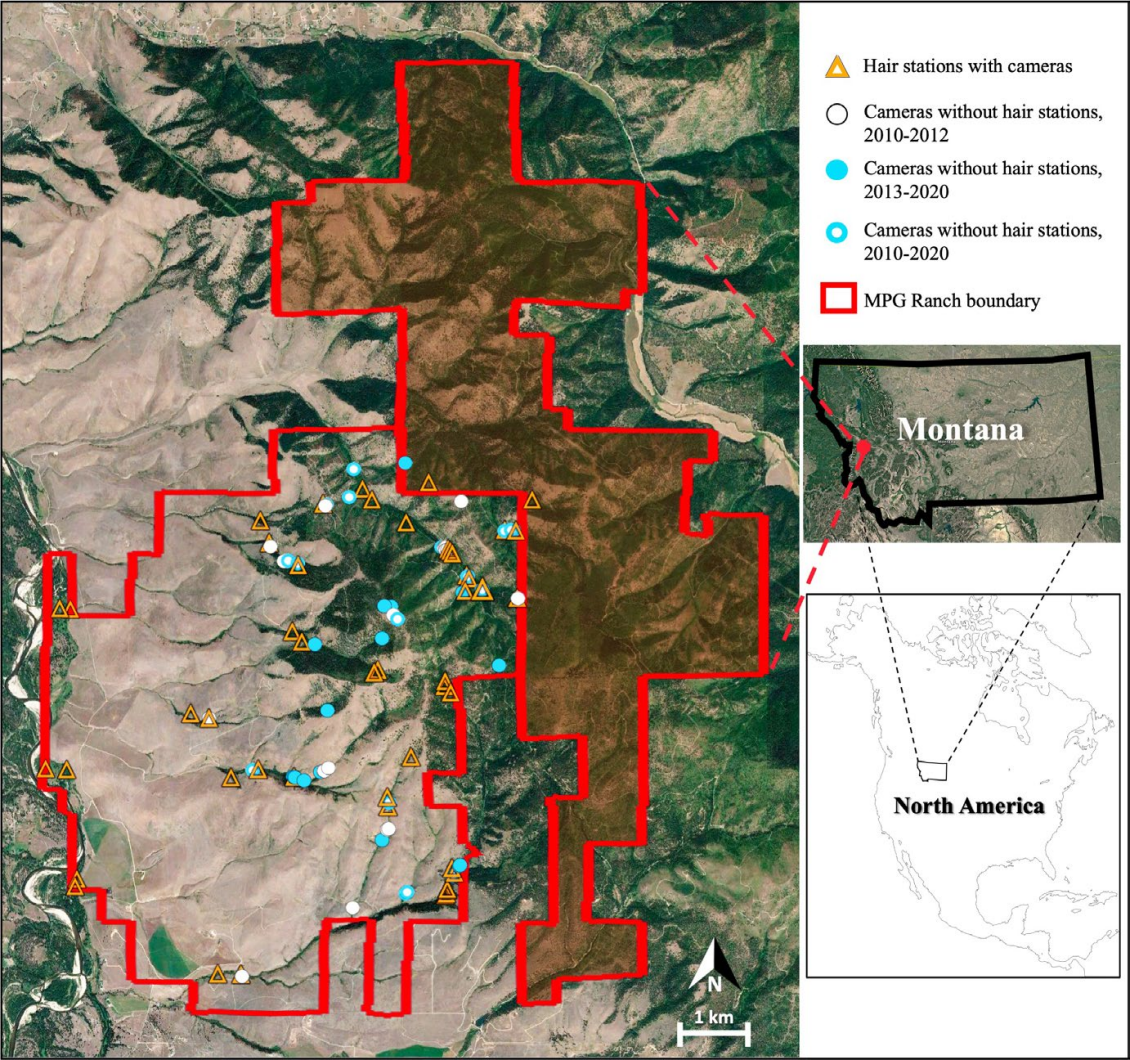
MPG Ranch, a 6,191‐ha conservation property in the Northern Sapphire Mountains in western Montana. The unshaded and shaded portions of MPG Ranch were purchased in 2009 and 2016, respectively. Satellite image provided by Google Earth

We conducted our study on MPG Ranch (46°42’26”N, 114°00’16”W), a 6,191‐ha conservation property located in the Northern Sapphire Mountains in western Montana, USA (Figure 1). The climate is temperate with snowy winters lasting ~5 months and sunny summers lasting ~3 months. Although elevations range from 966–1833 m, most of our research was conducted at the higher elevations where dominant tree species include Douglas fir (*Pseudotsuga menziesii*), Ponderosa pine (*Pinus ponderosa*), subalpine fir (*Abies lasiocarpa*), and quaking aspen (*Populus tremuloides*; Durham et al., 2017). Plants on the landscape that bears consume include cherry (*Prunus virginiana*, *P*. *emarginata*), serviceberry (*Amelanchier alnifolia*), huckleberry (*Vaccinium globulare*), arrowleaf balsamroot (*Balsamorhiza sagittata*), hawthorn (*Crataegus* spp.), sweet cicely (*Osmorhiza* spp.), cow parsnip (*Heracleum lanatum*), thimbleberry (*Rubus parviflorus*), strawberry (*Fragaria* spp.), currant (*Ribes* spp.), horsetail (*Equisetum* spp.), clover (*Trifolium* spp.), and other forbs and grasses (*Poaceae* spp.). Other large mammals on MPG Ranch include elk (*Cervus canadensis*), white‐tailed deer (*Odocoileus virginianus*), mule deer (*Odocoileus hemionus*), and mountain lions (*Puma concolor*). Prior to 2009, black bear hunting had occurred on the study site for decades. Beginning in 2009, black bear hunting was prohibited on the study site and on ~4000 ha of adjoining private lands. Black bear hunting occurred on nearby lands held in block management by The Nature Conservancy and on some nearby public lands. Deer and elk hunting occurred on the Ranch.

### 2.2 Video data

We used the following video camera systems to observe and detect bears: Stealth Cam model STC‐G42NG, Stealth Cam model STC‐DVIRHD (Grand Prairie, Texas, USA), Reconyx XR6 UltraFire (Grand Prairie, Texas, USA), Bushnell model 1197678 (Overland Park, Kansas, USA), and Browning models 8FHD‐P and BTC‐7A (Morgan, Utah, USA). During 2010, we placed video cameras at 23 stations to determine which areas were most frequented by bears. We added three video stations during 2011 and 33 additional video stations during 2012. We adjusted the location of some video stations in 2013 to maximize the detection of black bears and we removed four unproductive video stations. During 2013–2020, the number (*n* = 56) and placement of video stations were consistent. A station was defined as one or more video cameras aimed at a unique feature, such as a rub post, rub tree, wildlife trail, or water source. We often placed multiple video cameras at different angles at a single station to maximize individual identification. When multiple cameras captured an individual at a single station during a single event, we used video data from all cameras to identify the individual, but only one video was counted as a capture event. We placed cameras ~0.3–1.0 m off the ground to view black bear characteristics such as chest blazes, facial features (e.g., eyebrows, mustaches, etc.), genitals, and other body traits (e.g., coat color, body size, etc.). Cameras recorded for 1‐min intervals at up to 60 frames per second and were operational 24 h per day. We checked all video cameras every 2–3 weeks during summers 2010–2014 and replaced camera batteries at least 4 times annually. During 2015–2020, we checked cameras and replaced camera batteries every 6 weeks during March‐November, annually.

### 2.3 Hair DNA

In late fall 2012, we installed 28 hair collection systems. The hair collection systems included 12 rub trees, four rub posts, and 12 hair corrals. In 2013, we added four rub trees and four rub posts. For the rub trees, we wrapped barbed wire around the trunks of 16 trees that had already been established as rub trees by bears. We installed 12 rub posts, which were ~2‐meter‐high railroad ties wrapped with barbed wire. We constructed hair corrals using one strand of barbed wire (Ramsey et al., 2019; Sawaya et al., 2012) and placed corrals in locations that bears frequented. We distributed the hair collection systems over 3840 ha to maximize the number of individuals sampled. All hair collection systems were placed in view of multiple video cameras. The 12 hair corrals were removed in late 2014 because they were unproductive, but the 16 rub trees and eight rub posts remained in place throughout 2020. Also, three additional rub trees and six additional rub posts were installed during the years 2014–2016.

We used a non‐food scent lure at rub posts and hair corrals to attract bears. We did not use a lure (scent or food) at the 16 rub trees because bears were already using those trees for rubbing. Importantly, the initial goal of our camera‐based research was to document individual bear behaviors, so video and hair stations were placed in areas that were known to be frequented by bears, rather than in a grid pattern (Kendall et al., 2016; Laufenberg et al., 2018).

We defined a hair sample as all the hairs found on one barb of the barbed wire at the moment of hair collection. We partitioned each rub tree and rub post into 12 sections and documented in which section of the rub tree or rub post each hair sample was collected. We collected hair samples and rebaited rub posts and hair corrals with a non‐food scent lure every 2–3 weeks during summers 2013–2014 and every 2–3 weeks during March‐November, 2015–2020. We flame‐sterilized hair‐trap barbs between collections and dried hair samples in paper envelopes and stored them with silica desiccant.

We cleaned all hair samples of debris and we placed hairs with visible follicles in tubes for DNA extraction using the DNeasy^®^ Blood and Tissue kit (QIAGEN, Valencia, CA, USA). All DNA extractions were performed at either MPG Ranch or the U.S. Forest Service Rocky Mountain Research Station (RMRS), Missoula, MT. RMRS performed all individual identity analysis on purified extracts using a panel of nine microsatellite loci, including G1A, G10D, G10B (Paetkau & Strobeck, 1994), G10H, G10J, G10L, G10P, G10X and UarMu59 (Paetkau & Strobeck, 1998), plus one sex identification locus, SRY (Carmichael et al., 2005). We initially genotyped all DNA samples from hair in duplicate. Samples that produced inconsistent genotypes were re‐extracted and amplified three to six additional times. If a sample continually failed to produce a high‐quality genotype, it was removed from further analyses. To identify potential genotyping errors such as false positives or allelic dropout, RMRS ran all resulting genotypes through two error checking programs, DROPOUT (McKelvey & Schwartz, 2005) and Micro‐checker (Van Oosterhout et al., 2004). Results indicated that the final genotypes were free of errors. The probability of identity (i.e., the probability that two individuals drawn randomly from the population would have the same genotype at these loci) was 4.86 × 10^−11^. The probability of identity for siblings was 1.55 × 10^−4^. RMRS calculated both of these statistics using GenAlEx (Peakall & Smouse, 2006).

### 2.4 Identifying individuals

Using methods described by Ramsey et al. (2019), we used genetic‐based individual identifications to inform video capture‐based individual identifications. Generally, we separated videos of black bears from videos of other wildlife and chronologically sorted videos. For each video capture of a bear rubbing a rub feature (e.g., rub post or tree), we documented in which of the 12 sections of the rub feature the bear rubbed. Before genetic data were evaluated, one researcher meticulously scrutinized all video data and assigned an individual identification to each video capture using a combination of distinguishing bear characteristics, including permanent traits, individual behaviors, and temporary traits.

**FIGURE 2:**
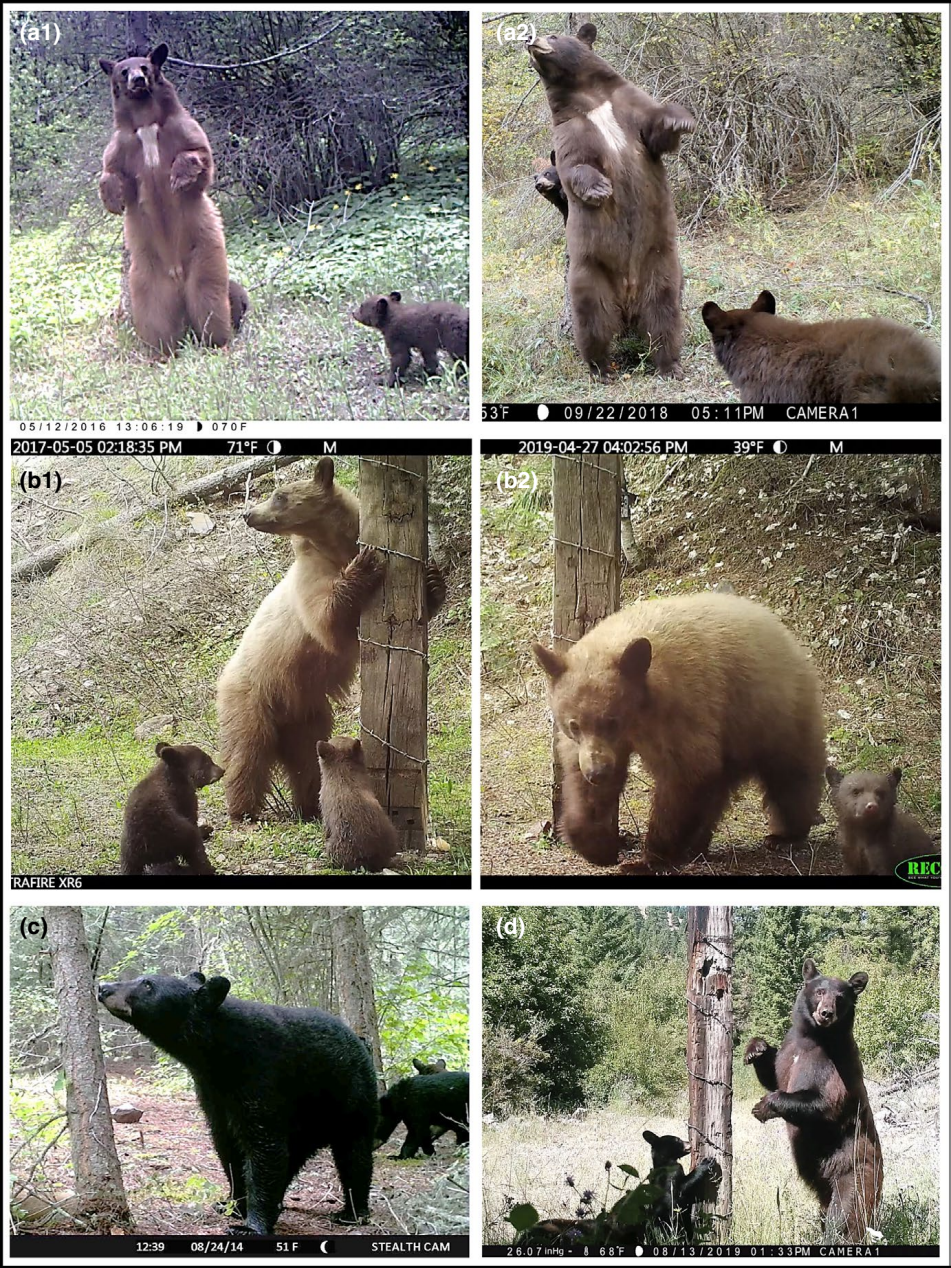
Examples of American black bear (*Ursus americanus*) mother–offspring video captures: (a1) adult female F7 with cubs F14 and M16 in 2016, (a2) adult female F7 with cubs F18 and U3 in 2018, (b1) adult female F6 with cubs F15 and M20 in 2017, (b2) adult female F6 with cub M31 in 2019, (c) adult female F1 with cubs F11 and M12 in 2014, and (d) adult female F11 with cubs F19, F21, and M33 in 2019

Permanent bear traits included blazes (Figure 2), ear notches, relative ear size, profile snout shape, coat color (Figure 2), eyebrow color, snout and head shape, snout mustaches, snout scars and lines, snout color, bare spots and scars, temporalis and masseter size, female or male genitals, and shoulder humps (Table 1). Individual behaviors included gait type, swim technique (e.g., one bear had a specific swimming style), etc. Temporary traits included wounds, coat‐shedding patterns during a given period, coat color in sunlight, wet coats, coat‐shedding across periods, burr presence, general weight gain and uneven weight gain, and nipple visibility. For example, individuals with facial wounds were relatively easy to identify and track as they healed through time. Also, most bears shed their winter coats in unique patterns. For example, some bears lost fur around their sides first, while others lost fur around their rumps first (for extended visual examples of bear traits, see Ramsey et al., 2019).

**TABLE 1:** Examples of permanent traits of individual American black bear (*Ursus americanus*) mothers and offspring, whose encounter histories were included in models of offspring annual apparent survival, on MPG Ranch during 2010–2020

Bear ID	Year born	Sex	Blaze	Ears	Eye brows	Mustache	Coat color	Snout	Other
F1	?	F	High dot				Black		
F2	2010	F					Dk. brown	Long	Distinct coat pattern
F3	?	F				Yes	Cinnamon		Distinct tail
F4	?	F		Pointed			Lt. brown	Wide	Stripe down back
F5	2011	F				Yes	Black		
F6	2011	F					Blonde		
F7	2012	F	Y‐shaped		Yes		Cinnamon		
F8	2012	F	hourglass				Cinnamon		
F9	2013	F					Dk. brown		
F11	2014	F	High dot			Yes	Dk. brown		
F12	2015	F	Thin stripe		Yes		Lt. brown		Sm. rumple
F13	2016	F					Brown		
F14	2016	F	Square			Yes	Lt. cinnamon		
F15	2017	F	Teardrop			Yes	Dk. brown		Small size
F16	2017	F					Brown		
F17	2017	F		Notch	Yes		Lt. brown		Paddle‐shaped tail
F18	2018	F					Dk. brown		
F19	2019	F				Yes	Black		
F20	2019	F					Lt. brown		
F21	2019	F	Three dots			Yes	Brown		
F22	2019	F					Brown		
M1	2010	M					Dk. brown	Long	Sm. rumple
M8	2013	M					Dk. brown		
M11	2014	M					Dk. brown		
M12	2014	M	Low dot	2 frostbitten			Black		
M13	2015	M					Brown		
M16	2016	M			Yes	Yes	Lt. cinnamon		
M20	2017	M		1 frostbitten			Blonde		
M22	2017	M					Brown		Distinct coat pattern
M23	2017	M				Yes	Dk. brown		
M31	2019	M					Brown		Pink nose
M33	2019	M	Medium oval				Brown		
M34	2019	M					Brown		
M39	2019	M				Yes	Black		
M42	2019	M				Yes	Brown		
M45	2020	M					Brown		
UB	2010	U					Black		
U1	2014	U					Brown		
U2	2017	U					Brown		
U3	2018	U					Dk. brown		
U4	2019	U					Black		
U6	2020	U				Yes	Brown		
U7	2020	U				Yes	Brown		
U8	2020	U				Yes	Brown		

Frequent and regular video captures of individuals aided individual identification. For example, we estimated that Bear F7 was video‐captured 234 times during 2013–2020 (Table 2). Bear F7 was easily identifiable by a distinct chest blaze (Figure 2a1 and a2). Regular captures of individual bears allowed us to track gradual changes and temporary marks. This helped us minimize misidentification errors associated with camera data due to changes in natural marks (Yoshizaki et al., 2009).

**TABLE 2:** The number of video and genetic capture events of American black bear (*Ursus americanus*) mothers and offspring on MPG Ranch in western Montana, 2013–2020

Bear ID	Mother	2013	2014	2015	2016	2017	2018	2019	2020	Total captures
F1		60	70	66	31					227
M1	F1	19	38	9	32	8	20	12	13	151
F2	F1	130	101	145	127	112	156	133	194	1098
F3		11								11
F4		12	37	17	44	42	72	63	42	329
F5	F4	7	3	5						15
F6	F4	55	74	86	134	131	121	73	82	756
F7	F3	32	31	17	35	34	44	20	21	234
F8	F3	5			13	12				30
F9	F4	14	15	1	9	16	2	15	16	88
M8	F4	15	7*							15
M11	F2		104	65	40					209
U1	F2		1							1
M12	F1		69	53	100	14				236
F11	F1		69	80*	83	95	91	54	84	476
M13	F4			17	29	13				59
F12	F4			17	19	6	6	1	4	53
F13	F2				23*					23
F14	F7				21	59*	16	7	14	117
M16	F7				27	5				32
M20	F6					127	3			130
F15	F6					119	45*	87	42	293
U2	F2					2				2
M22	F2					116	115*	69	2	302
F16	F2					47				47
F17	F4					43	23*	56	98	253
M23	F4					36	34*	47		117
F18	F7						40	67	19	126
U3	F7						34	2		36
M31	F6							23		23
F19	F11							46	37*	83
M33	F11							47	63*	110
U4	F2							8		8
M34	F2							121		121
F20	F2							116*		0
F21	F11							43	48*	91
F22	F4							55	4	59
M39	F9							14	11*	14
M42	F9							12	8*	12
M45	F6								76*	76
U6	F7								14	14
U7	F7								18	18
U8	F7								12	12

Note

Yellow shading: video captures of cubs. Orange shading: video captures of yearlings where yearlings were identified with mothers. Red shading: video captures of yearlings where yearlings were identified without mothers. Green shading: video captures of 2+ year olds where genetic data were concurrently collected. Gray shading: video captures of 2+ year olds where genetic data were not concurrently collected. Asterisk: video captures of cubs or yearlings where genetic data were concurrently collected

All video captures of cubs were collected at camera stations, except one. Bear U1 (Table 2) was not video‐captured at a station but was filmed by a researcher over a period of five hours with its mother (Bear F2) during May 4–6, 2014. We feel confident that Bear U1 was the cub of Bear F2 because Bear F2 was easily identifiable owing to her unique coat pattern.

We identified video‐captured cubs during their first year and we tracked cubs that remained in the study area through time. Cubs that were video‐captured were always with their mothers, so we used traits of mothers to help identify cubs. Some cubs had unique traits, which we also used for cub identification. Cubs that were recaptured the following year as yearlings were almost always video‐captured with their mothers at least once before the family break‐up, which helped us identify individual yearlings. The two exceptions were yearlings Bear F15 and Bear M20, offspring of Bear F6. Bear F15 had a distinct blaze and Bear M20 had a frostbitten right ear and a very light‐colored coat, making both bears easy to identify in 2018 without their mother. As cubs transitioned into yearlings, they experienced shifts in coat and morphological appearance. We documented the shifted traits for individual yearlings during video captures of yearlings with their mothers. Subsequently, we used the shifted individual traits of yearlings to help identify yearlings throughout their second year and as they transitioned into older age classes. We also used the date stamp on each video to help determine individual identities, as some yearlings captured in spring can almost double in size by fall.

After we identified individuals using video data, genetic analyses based on hair samples were used to cross‐reference and inform individual identifications. We used the cross‐referencing method only when a bear deposited hair DNA. Some individuals were video‐captured only a few times and never deposited hair so we could not use genetic data to cross‐reference identifications for those individuals. All but two of those bears had distinct traits, which we used to identify them. For example, Bear M19 had a distinct chest blaze and white lips, Bear M25 had a distinct blaze, Bear F3 was a mature female with a distinct tail, and Bear F10 was a mature female with a cinnamon‐colored coat and a distinct floppy ear tear in her right ear (Reynolds‐Hogland et al., 2022). Two bears (Bears M18 and M27) were identified based on the process of elimination (Reynolds‐Hogland et al., 2022). Although unlikely, we might have misidentified these two individuals.

To cross‐reference video data with hair samples, we were able to match bears captured via video to their hair samples for many capture events when there was only one bear observed via video and one hair sample collected during a sampling period. When 2+ bears left hair during a sampling period, we matched many bears observed via videos to their hair samples based on which section of the rub tree or post each bear rubbed and in which section of the rub tree or post hairs were collected. If two bears rubbed in the same section of a rub tree or post, the general result was a mixed genetic sample, which did not successfully genotype. We documented sex for individual bears when genetic‐based sex identifications were available, and/or when genitals or engorged nipples were visible on video captures.

### 2.5 Estimating individual identification accuracy

During some years, we captured some individuals at hair stations via only video cameras. Also, video stations placed at wildlife trails or water sources did not include hair collection systems. Therefore, we estimated the individual identification accuracy rate using only video data. To do this, we compared identifications of individuals based on only video data with genetic‐based identifications using bear hair concurrently collected at hair stations equipped with video cameras (Ramsey et al., 2019; Reynolds‐Hogland et al., 2022). Our identification accuracy assessment was a blind test because we first identified bears via video before genetic data were analyzed (i.e., the original video‐based identifications were uninformed by genetic data). A genetic identification‐video identification set (hereafter named genetic‐video set) was defined as one genetic identification and all blind video identifications from the same station within a sampling period. Because some bears left multiple (i.e., redundant) hair samples at the same time and location, we included only one successfully genotyped hair sample per video‐capture event to prevent inflation of the identification accuracy estimate. If the video‐based identification correctly matched the genetic‐based identification, the genetic‐video set was considered accurate. If the video‐based identification did not correctly match the genetic‐based identification, the genetic‐video set was considered inaccurate. Cameras generally recorded hair depositions by bears, but they failed to video‐document bears depositing hair if camera batteries died, memory cards were full, or if other mechanical failures occurred. Hair samples deposited by bears that were not concurrently video‐documented were censored from individual identification accuracy rate analyses.

### 2.6 Genetic data collected during live‐capture in 2020

In summer 2020, we began a pilot study for another research project that included live‐capture and collaring black bears on the study site. We collected blood and hair samples from captured bears, which we used to determine genetic identity and paternity and to test maternity determinations based on video observations. We immobilized non‐cub bears using telazol (Zoetis, Parsippany, New Jersey, USA), administered with a dart pistol or pole syringe. Each immobilized bear was weighed, sexed, PIT‐tagged, measured, and ear‐tagged. For cubs and non‐yearling bears, we pulled a premolar for age determination via cementum analysis (Matson's Lab, Manhattan, MT, USA). We attached Vertex Plus GPS collars (Vectronic Aerospace, Berlin, Germany) to bears that weighed ≥55 pounds. Each collar band bore unique symbols to aid in bear identification upon recapture. We collected blood from most captured individuals and placed a few drops on FTA blood collection cards. In the lab, we punched three 3.00 mm holes into the blood‐soaked filter paper using a Harris Micro‐Punch and DNA was extracted from the FTA paper using the DNeasy^®^ Blood and Tissue kit (QIAGEN, Valencia, CA, USA). Individual identity analyses were performed on purified extracts using the same methods as that described using hair DNA. Throughout the field season, we followed the University of Montana's Covid‐19 Guidelines for Field Research. Our protocol for handling bears was approved by the Institutional Animal Care and Use Committee of Montana Fish Wildlife and Parks (IACUC #: FWP02‐2020).

### 2.7 Pedigree

We created a population‐level pedigree with individual birth years for wild black bears in our study site. For many wild species, including black bears, parental care by the mother makes it easy to identify maternity based on observation (Städele & Vigilant, 2016). We used video observations of family units to determine maternity for individual cubs and sibling relationships when more than one cub was in a litter. For all but one mother–offspring pair, offspring were cubs at first video observation. The one exception was a mother–yearling pair that we observed before the family break‐up in 2011. When genetic data were available for both mother and offspring, we also determined maternity across 10 loci using exclusion conducted by hand and subsequently using the program Cervus 3.0 using the strict 95% confidence criteria to assess those relationships (https://cervus.software.informer.com/3.0/, accessed 30 March 2021). We used the same method to determine paternity. Not all adult males provided genetic data, so we did not sample all candidate fathers. However, every male for which we had genetic data was tested for possible paternity of every genetically identified bear in our study site. In 2016, we observed the death of one cub via video data and we obtained her genetic identity based on tissue samples that we collected during the necropsy.

### 2.8 Litter size, interlitter interval, primiparity, natality, and fecundity

We estimated litter size based on the earliest observations of complete litters after den emergence (Miller, 1994; Miller et al., 2003). We estimated interlitter interval for all females that produced at least two litters during our study period. Two reproductively successful females disappeared midway through our study, so we included interlitter interval data for those two females only during the period when each was present in the study area. To estimate primiparity, we included only females of known age (i.e., females we had tracked since they were cubs). We estimated the mean natality (number of cubs/female/year) of monitored females that were ≥4 years old. We estimated the mean annual fecundity rate (*m*) of monitored adult females following Garrison et al. (2007). Although we knew sex for most cubs in most litters, we did not know the sex for all cubs so we assumed the sex ratio of litters was 50:50. Thus, *m* for each year *x* was calculated as the number of female cubs born during year *x* (total number of cubs/2) divided by the number of adult females monitored during year *x*.

### 2.9 Cub detection rate

During some video‐capture events of family groups, mothers may have been in view of cameras while cubs were not. Therefore, we estimated the cub detection rate by calculating the proportion of video‐capture events during which all littermates of each multi‐cub litter were observed together during year 1. To estimate cub detection rate, we included only multi‐cub litters that were known to have survived year 1 so that the number of cubs that should have been present during video captures was known.

### 2.10 Effects of maternal identity on offspring annual apparent survival

#### 2.10.1 Using combined genetic and video data

To model offspring apparent survival, we included video and genetic data collected during 2013–2020 because the number (*n* = 56) and placement of video stations were consistent during this period. During 2013–2020, we documented the number of capture events for each offspring, each year. For example, if a cub was born in 2013, we documented the number of capture events during the cub's first year and all subsequent years, through 2020 (Table 2). We created encounter histories for each offspring based on a 1‐year time interval by collapsing total capture events per offspring, per year into single data points (“1” if offspring *i* was captured, or “0” if offspring *i* was not captured, during year *x*). We used the Cormack‐Jolly‐Seber model (CJS; Lebreton et al., 1992) in Program Mark (White & Burnham, 1999) to estimate the annual apparent survival of offspring (the probability that an offspring individual is alive and remains on the study area and hence is available for recapture; *φ*) and offspring recapture probability (*p*). To evaluate the effect of maternal identity on offspring *φ*, we grouped offspring individuals into maternal identity groups. There were eight maternal identities (i.e., eight different mothers) and 40 encounter histories (i.e., 40 offspring), so it was important to minimize the total number of estimable parameters. We also evaluated models that included time‐dependent and constant *φ* and p. We considered the intercept‐only model (*φ* {.} *p* {.}) to be the null model. We used the CJS model rather than other more complex models because the CJS model had the least number of estimable parameters (only *φ* and *p*).

Apparent survival was bounded between 0 and 1, so we used the logit link to develop models of *φ*. We evaluated the goodness‐of‐fit of the saturated model using a bootstrap approach with 1000 simulations (Franklin et al., 2004). The saturated model was defined as the model for which the number of parameters equaled the number of data points or data structures (Cooch & White, 2002). For our data, the saturated model was *φ* (maternal identity) *p* (maternal identity). We estimated the overdispersion parameter (c) and, in the case of overdispersion, we adjusted c‐hat accordingly. We used Akaike's (Akaike, 1973) information criterion adjusted for sample size (QAIC_c_) to rank models in terms of their ability to explain the data. Models with Δ QAIC_c_ values <2.0 were considered to have substantial support (Burnham & Anderson, 2002). We evaluated Akaike weights for each model (Burnham & Anderson, 1998).

#### 2.10.2 Using only genetic data

We performed a second offspring *φ* analysis using only genetic data and compared those model rankings and parameter estimates with model rankings and parameter estimates derived from combined genetic and video data. We created encounter histories for each genetically identified bear based on a 1‐year time interval, by collapsing total genetic captures (from hair DNA, blood DNA, and tissue DNA) per bear, per year into single data points for each bear and year from 2013 to 2020. We used the CJS model to estimate *φ* and *p*, and to evaluate the effects of maternal identity on *φ*. We considered the intercept‐only model (*φ* {.} *p* {.}) to be the null model, we evaluated the goodness‐of‐fit of the saturated model, we used QAIC_c_ to rank models, and we considered models with Δ QAIC_c_ values <2.0 to have substantial support.

## 3 RESULTS

### 3.1 Video and hair data

During 2010–2020, we documented 9,241 video‐capture events of an estimated 94 individual black bears (54 M: 29 F: 11 Unknown gender; Table 3). During 671 capture events (7%), the individual bear was not identifiable because the bear was obstructed by darkness, the bear was too far from the video camera, or the bear was otherwise not in view. Total trap effort throughout the entire study duration was 145,792 camera trap days.

**TABLE 3:** (a) The number of hair DNA stations, hair samples, individual American black bears (*Ursus americanus*) detected, and number of cubs and yearlings detected via hair DNA, and (b) the number of video camera stations, capture events, individuals detected, and yearlings and cubs detected on MPG Ranch each year, 2010–2020

(a) DNA data					
Year	No. hair stations	No. hair samples	No. individuals detected	No. yearlings detected	No. cubs detected
2010	–	–	–	–	–
2011	–	–	–	–	–
2012	28	36	7	0	0
2013	36	149	10	0	0
2014	44	90	12	1	0
2015	30	35	4	1	0
2016	32	103	15	0	0
2017	33	82	12	0	0
2018	33	127	12	5	0
2019	33	197	15	1	1
2020	33	308	25	5	1
Total no. unique individuals		54			

(b) Video data					
Year	No. video stations	No. capture events	No. individuals detected	No. yearlings detected	No. cubs detected
2010	23	24	4	0	2
2011	26	60	11	4	2
2012	59	370	15	3	2
2013	56	620	20	2	2
2014	56	868	18	2	4
2015	56	804	18	3	2
2016	56	1166	24	3	3
2017	56	1274	28	2	7
2018	56	1302	31	8	2
2019	56	1531	41	5	10
2020	56	1390	42	7	4
Total no. unique individuals		94			

During 2013–2020, we collected 1091 black bear hair samples and successfully extracted DNA from 1025. Samples were assumed to be black bears, though as we did not perform DNA species identification due to the focus of the study, some samples were likely from nontarget species. Hair samples that did not successfully genotype either had bear hair that did not contain quality DNA or the samples were of non‐bear species. We successfully genotyped 468 (46%) of those hair samples, from which we identified 54 unique individual bears (35 M: 18F: 1 Unknown gender; Table 3). We observed eight mothers via video. Of these, seven left hair DNA from which we determined genetic identifications.

The amount of time that was required to set up camera and hair stations was ~175 h. We spent an additional ~255 h (~32 staff days) visiting stations to switch out SD cards and to collect hair samples, annually. Analyzing video data and cross‐referencing video captures with genetic captures required ~400 h (~50 staff days), annually.

### 3.2 Individual identification accuracy

We collected 210 hair samples that successfully genotyped of the 41 bears that were included in our *φ* analyses. Of the 210 hair depositions by bears, 203 (97%) were concurrently video‐documented. After removing redundant hair samples, our sample size for estimating individual identification accuracy was 134 successfully genotyped hair samples matched with 134 video‐capture events. Of the 134 genetic‐video sets, we accurately matched the video identification with the genetic identification 130 times (97%). Previously, we also demonstrated that we accurately identified yearling bears across time (Reynolds‐Hogland et al., 2022). In all cases where a bear was genetically captured as a yearling and subsequently recaptured as either a yearling or 2+ year‐old bear (*n* = 38 recaptures of six yearlings), our video‐based identifications correctly matched the genetic‐based identifications.

### 3.3 Genetic data from live‐capture during 2020

We live‐captured 13 individuals during our pilot study in 2020 and determined genetic identities for all 13 from blood or hair DNA collected during the handling process. Eight of those 13 bears had previously left hair DNA so we already knew their genetic identities. Three live‐captured bears were yearlings (with distinct traits) that we had previously observed with their mothers (who had distinct traits), but who had not previously left hair DNA. For all three, we obtained genetic identities, confirmed the mother–offspring relationships that we had estimated based on video observations, and evaluated paternity using DNA analyses. Two live‐captured bears were subadult males that we had neither previously observed via video nor detected via hair DNA on our study site. Neither subadult male was the offspring of any female or male for which we had genetic identities.

### 3.4 Pedigree

When constructing wild pedigrees, founders and immigrants are assumed to be unrelated and non‐inbred (Pemberton, 2008; Städele & Vigilant, 2016). We evaluated this assumption by assessing all possible parent–offspring relationships among all genetically identified bears. None of the genetically identified males sired either of the two genetically identified founding females (Bears F1 and F4), no genetically identified female offspring mated with their fathers, and no genetically identified male offspring mated with genetically identified females. We did not evaluate other possible relationships (e.g., cousins). However, mammals show a tendency towards male‐biased dispersal (Dobson, 1982), the evolution of which may have been driven, in part, by inbreeding avoidance (Handley & Perrin, 2007; Pusey, 1987). For black bears, most male offspring disperse before they reach reproductive age (Costello, 2010; Schwartz & Franzmann, 1992), which should have prevented or minimized inbreeding of bears in our study (but see Kendall et al., 2016).

**FIGURE 3:**
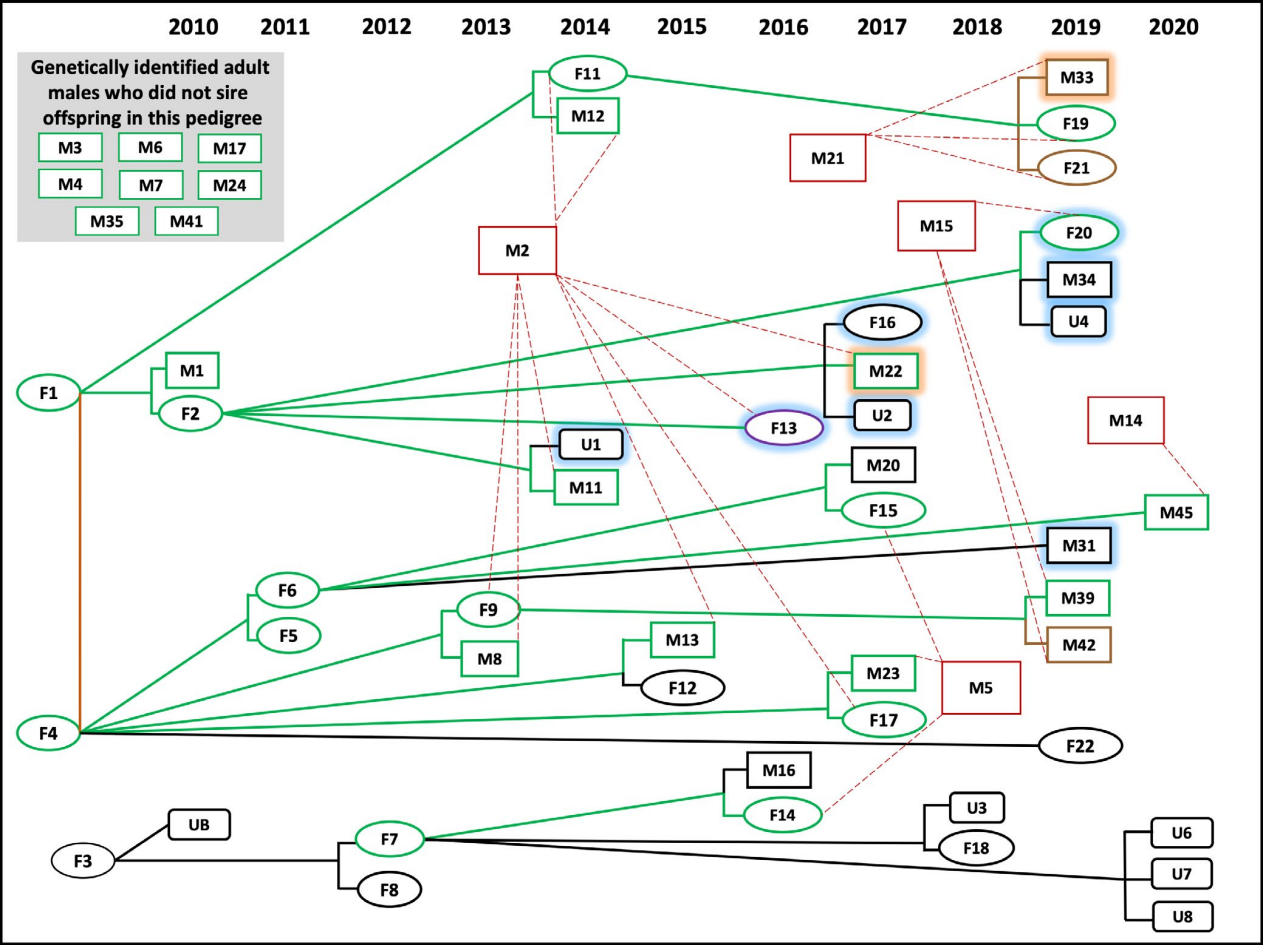
Age‐specific, population‐level pedigree of wild American black bears (*Ursus americanus*) on MPG Ranch in western Montana, 2010–2020. Ovals represent females, rectangles represent males, and rounded rectangles represent unknown gender. Green lines connecting green symbols represent mother–offspring relationships observed via video and confirmed by genetics via hair DNA. Brown symbols connected to green symbols by green lines represent mother–offspring relationships observed via video and confirmed by genetics via blood DNA during live‐capture in 2020. Dark pink symbol connected to green symbol by green line represents mother–offspring relationship observed via video and confirmed by genetics via tissue sample of deceased cub during necropsy. Red symbols represent adult males who sired offspring, connected by red dashed lines representing father–offspring relationship determined genetically. Black symbols represent individuals who did not leave genetic data and black connecting lines represent mother–offspring relationships that were captured via video only. The orange line represents a mother–offspring relationship documented by genetics only. Blue shading around a symbol represents a bear that died when it was a cub. Orange shading around a symbol represents a collared bear that died of natural causes while it was a yearling or subadult

Although we observed 94 individual bears during 2010–2020, the population‐level pedigree included only individuals for whom we documented relatedness (*n* = 49; Figure 3). In addition to the 49 related individuals, we obtained genetic identities for eight adult males who did not sire genetically identified offspring (Figure 3, top left). We did not collect genetic data for all offspring, so it is possible that one or more of these eight adult males sired offspring in our study area.

The pedigree included 42 mother–offspring pairs. We identified 41 mother–offspring pairs via video. One mother–offspring pair was determined via only hair DNA (Figure 3, Bears F1 and F4). Both of these females were adults at the beginning of our study, so we could not be certain which was the mother and which was the daughter. In every case where hair DNA data were available for both mother and offspring (*n* = 20 mother–offspring pairs), genetic analyses confirmed the presumed mother–offspring relationship based on video observation (Figure 3, green lines connecting green‐outlined symbols). In addition, three of the mother–offspring pairs identified via video were genetically confirmed via blood DNA collected from three yearlings during live‐capture in 2020 (Figure 3, brown‐outlined symbols). One mother–offspring pair identified via video in 2016 was genetically confirmed postmortem via tissue sampling of the deceased cub during necropsy (Figure 3, pink‐outlined symbol). Seventeen mother–offspring pairs identified via video were not identified via genetics (Figure 3, black‐outlined symbols).

Of the 42 mother–offspring pairs in 22 litters included in the pedigree, we documented 21 litters via video data. Had we used hair DNA alone, we would have identified only 15 litters. Of the 42 mother–offspring pairs, 40 offspring were first observed via video during their cub year. Of these 40 cubs, 36 were observed during 2013–2020 when hair stations were active. Yet, only two cubs were detected via hair DNA during 2013–2020 (Table 3a). Every mother–offspring pair observed via video during 2013–2020 was observed at least once at a hair station when the offspring was a cub, but cubs of the year generally did not rub or otherwise leave hair at hair stations. The two exceptions were Bears F20 and M45, both of whom frequently rubbed on rub posts when they were <1. Based on video data, we determined that eight offspring died during their first year (Figure 3, symbol outlines shaded in blue). Two bears that wore GPS collars as part of our live‐capture study in 2020 died of natural causes (Figure 3, symbol outlines shaded in orange).

Beginning in 2013, we were able to determine paternity for all genetically identified offspring. We observed 13 genetically identified adult males in our study site, only five sired offspring in the pedigree. One adult male (Bear M2) sired nine cubs in seven litters with three different mates (Figure 3). Three other adult males (Bears M5, M15, M21) sired three cubs each and one adult male (Bear M14) sired one cub. We did not collect genetic data for several offspring so the number of sired cubs for each individual adult male could have been higher than what we reported. We documented multiple paternity for one litter: Bear M2 sired Bear F17 and Bear M5 sired Bear M23 (Figure 3).

### 3.5 Litter size, interlitter interval, primiparity, natality, and fecundity

During 2010–2020, the mean litter size was 1.95 (95% CI: 1.65 2.3). We observed 13 interlitter intervals for six of the eight females that bore cubs; most litters (*n* = 10) were produced at 2‐year intervals, two litters were produced at 1‐year intervals, and one litter was produced at a 4‐year interval (Table 4). The mean interlitter interval was 2.0 years (95% CI: 1.57, 2.43). For the 14 litters for which we knew the gender of all littermates, the sex ratio of cubs was 0.86:1 (12 M:14F).

**TABLE 4:** Age of first litter, number of cubs per litter, interlitter interval, total cubs, total litters for monitored adult (≥4 years old) females, and mean annual fecundity rate of monitored adult female American black bears (*Ursus americanus*) on MPG Ranch during 2010–2020

Bear ID	Year born	Year first litter	Age at first litter	Number of cubs in litter										
				2010	2011	2012	2013	2014	2015	2016	2017	2018	2019	2020
F1	?	?	?	2	–	–	–	2	–	–	X	X	X	X
F3	?	?	?	1	–	2	–	X	X	X	X	X	X	X
F4	?	?	?		2	–	2	–	2	–	2	–	1	–
F2	2010	2014	4					2	–	1	3	–	3	–
F5	2011	NA	NA						–	X	X	X	X	X
F7	2012	2016	4							2	–	2	–	3
F8	2012	NA	NA							–	–	X	X	X
F6	2011	2017	6						–	–	2	–	1	1
F9	2013	2019	6								–	–	2	–
F10	2014	2019	5									–	3	–
F11	2015	NA	NA										–	–
F14	2016	NA	NA											–
Total cubs^a^				3	2	2	2	4	2	3	7	2	10	4
Total litters				2	1	1	1	2	1	2	3	1	5	2
Adult females monitored^b^				2	3	3	3	3	5	6	6	6	7	8
Annual fecundity^c^				0.75	0.33	0.33	0.33	0.67	0.20	0.25	0.58	0.17	0.71	0.25

Note

X = female not observed during year.

^a^ Total number of cubs (males and females) born to monitored females.

^b^ Total number of monitored females of reproductive age; ≥4 years old.

^c^ Calculated as the no. F cubs (No. Cubs/2) divided by no. monitored adult females.

Of the 12 adult females we monitored, eight produced cubs during the study period. Of these, two females produced cubs in 2010 and one female produced cubs in 2011, so we could not determine the age of primiparity for these three adult females. Therefore, our estimate of the age of first reproduction was based on five adult females: two first produced cubs at age 4, one first produced cubs at age 5, and two first produced cubs at age 6 (Table 4). The mean age of primiparity was 5 years (95% CI: 3.76, 6.24). The mean natality of female bears ≥4 years old was 0.59 (95% CI: 0.27, 0.91). The mean fecundity rate varied across years from 0.17 to 0.75 (Table 4), and the mean fecundity rate over the study period was 0.42 (95% CI: 0.27, 0.56).

### 3.6 Cub detection rate

To estimate the cub detection rate, we included video observations of 25 cubs in 12 multi‐cub litters. Eleven litters contained two cubs and one litter contained three cubs. We captured the 25 cubs 419 times on video during 2010–2020. For three of the two‐cub litters, both littermates were always video‐observed together with their mother (Littermates F5 & F6, F7 & F8, M15 & F12; Table 5). For the other nine multi‐cub litters, we occasionally video‐documented partial litters with their mothers. For all nine litters, the date of the first observation of a partial litter (with their mother) always occurred either after or on the same date that we first observed the entire litter together (with their mother; Table 5). The proportion of video captures of family groups in which all littermates of multi‐cub litters were observed together was 0.93 (95% CI: 0.91, 0.94). Beginning in year 2013, littermates of multi‐cub litters were video‐captured together at least 12 times during year 1 (Table 5).

**TABLE 5:** The multi‐cub American black bear (*Ursus americanus*) litters that were included in estimates of cub detection rate on MPG Ranch during 2010–2020. First observations of single cubs alone in a litter occurred either after or on the same date as the first observation of the entire litter

Littermates	Year litter born	No. times entire litter observed	Date of 1st observation of entire litter	Date of 1st observation of single cub of litter	Date of last observation of entire litter
F2 & M1	2010	4	8/20/2010	8/20/2010	9/24/2010
F5 & F6	2011	1	9/6/2011	NA	9/6/2011
F7 & F8	2012	5	8/5/2012	NA	9/5/2012
F9 & M9	2013	14	5/30/2013	8/10/2013	10/27/2013
M13 & F10	2014	63	6/7/2014	8/2/2014	10/21/2014
M15 & F12	2015	17	7/6/2015	NA	9/23/2015
F14 & M18	2016	21	5/12/2016	7/17/2016	9/28/2016
M22 & F15	2017	116	4/22/2017	7/5/2017	10/12/2017
F17 & M25	2017	35	5/23/2017	8/1/2017	9/25/2017
F18 & U6	2018	34	6/4/2018	8/26/2018	9/29/2018
F19, M36, F21	2019	36	6/13/2019	6/13/2019	10/27/2019
M42 & M46	2019	12	9/1/2019	9/1/2019	10/16/2019

### 3.7 Effects of maternal identity on offspring annual apparent survival

#### 3.7.1 Using combined genetic and video data

Using combined genetic and video data, our sample size for modeling offspring *φ* was 40. The estimated value of c‐hat was 1.17, within the range for global model fit (Anderson et al., 1994; Lebreton et al., 1992), so we adjusted for overdispersion. Two models had Δ QAIC_c_ <2.00. The top‐ranked model included the effect of maternal identity on offspring *φ* and the second‐ranked model was the null model (Table 6a). The AIC_c_ weight for the top‐ranked model was 0.57, compared with 0.28 for the null model and 0.13 for the third‐ranked model. Models with the effect of time dependency on either *φ* or *p* ranked very low, indicating neither *φ* nor *p* varied by time during our study.

**TABLE 6:** (a) Model rankings of annual apparent survival (*φ*) of American black bear (*Ursus americanus*) offspring based on combined genetic and video data, on MPG Ranch in western Montana during 2013–2020. Δ QAIC_c_ = difference between model QAIC_c_ and lowest QAIC_c_. ω = QAIC_c_ model weight. k = number of estimable parameters. Deviance = measure of model fit. (b) Estimates of *φ* and recapture (*p*) based on the top‐ranked model, with SEs and 95% confidence intervals

(a)					
Model	Δ QAICc	ω	Model Likelihood	*k*	Deviance
*φ* (Maternity ID) *p* (.)	0.00	0.57	1.00	9	50.22
*φ* (.) *p* (.) Null model	1.41	0.28	0.50	2	67.46
*φ* (.) *p* (Maternity ID)	3.00	0.13	0.22	9	53.21
*φ* (t) *p* (.)	7.95	0.01	0.02	8	60.57
*φ* (.) *p* (t)	9.87	0.00	0.01	8	62.49
*φ* (Maternity ID) *p* (t)	10.88	0.00	0.00	15	45.48
*φ* (t) *p* (Maternity ID)	11.04	0.00	0.00	15	45.63
*φ* (Maternity ID) *p* (Maternity ID)	11.68	0.00	0.00	16	43.45
*φ* (t) *p* (t)	18.90	0.00	0.00	14	56.25

(b)					
Model	Parameter	Estimate	SE	LCI	UCI
*φ* (Maternity ID) *p* (.)	*φ* F1 offspring	0.96	0.04	0.73	1.00
	*φ* F2 offspring	0.39	0.15	0.16	0.68
	*φ* F3 offspring	0.92	0.09	0.55	0.99
	*φ* F4 offspring	0.89	0.06	0.71	0.96
	*φ* F6 offspring	0.68	0.21	0.24	0.93
	*φ* F7 offspring	0.81	0.14	0.42	0.96
	*φ* F9 offspring	1.00	0.00	0.99	1.00
	*φ* F11 offspring	1.00	0.00	0.99	1.00
	*p*	0.97	0.02	0.87	0.99
Null	*φ* All offspring	0.84	0.04	0.74	0.90
	*p*	0.96	0.03	0.85	0.99

Based on the top‐ranked model, estimated *φ* for offspring of Bear F2 (0.39; SE = 0.15; Table 6b) was significantly lower than that for offspring of Bear F1 (0.96; SE = 0.04), Bear F4 (0.89; SE = 0.06), Bear F9 (1.00; SE = 0.00), and Bear F11 (1.00; SE = 0.00). Estimated recapture probability was 0.97 (SE = 0.02).

#### 3.7.2 Using only genetic data

Using only genetic data, our sample size for modeling offspring *φ* was 24. The estimated value of c‐hat was 1.28, within the range for global model fit (Anderson et al., 1994; Lebreton et al., 1992), so we adjusted for overdispersion. One model had Δ QAIC_c_ <2.0 (Table 7a). The top‐ranked model was the null model. The AIC_c_ weight for the top‐ranked model was 0.97, compared with 0.02 for the second‐ranked model. All other models ranked very low and had zero model weight. Based on the top‐ranked null model, estimated *φ* was 0.82 (SE = 0.07) and estimated *p* was 0.64 (SE = 0.11; Table 7b).

**TABLE 7:** (a) Model rankings of annual apparent survival (*φ*) of American black bear (*Ursus americanus*) offspring based on only genetic data, on MPG Ranch in western Montana during 2013–2020. Δ QAIC_c_ = difference between model QAIC_c_ and lowest QAIC_c_. *ω* = QAIC_c_ model weight. *k* = number of estimable parameters. Deviance = measure of model fit. (b) Estimates of *φ* and recapture (*p*) based on the top‐ranked model, with SEs and 95% confidence intervals

(a)					
Model	Δ QAICc	*ω*	Model likelihood	*k*	Deviance
*φ* (.) *p* (.) Null model	0.00	0.97	1.00	2	58.21
*φ* (.) *p* (t)	7.49	0.02	0.02	8	49.08
*φ* (t) *p* (Maternity ID)	10.67	0.00	0.00	9	48.80
*φ* (t) *p* (.)	11.34	0.00	0.00	8	52.93
*φ* (.) *p* (Maternity ID)	16.42	0.00	0.00	9	54.55
*φ* (Maternity ID) *p* (.)	16.45	0.00	0.00	9	54.58
*φ* (t) *p* (t)	30.07	0.00	0.00	14	46.37
*φ* (Maternity ID) *p* (t)	34.68	0.00	0.00	15	45.42
*φ* (Maternity ID) *p* (Maternity ID)	45.62	0.00	0.00	16	50.27

(b)				
Parameter	Estimate	SE	LCI	UCI
*φ* All offspring	0.82	0.07	0.63	0.92
*p*	0.64	0.11	0.42	0.82

We estimated apparent survival, which was confounded with permanent emigration. Most male black bear offspring disperse when they are 1–3 years old (Costello, 2010; Schwartz & Franzmann, 1992), so the true survival of offspring on our study site may be higher than what we report.

## 4 DISCUSSION

We successfully reconstructed a population‐level pedigree of wild black bears with individual birth years using multiple data sources. Importantly, 21 of the 42 mother–cub pairs that we documented would have gone undetected had we used only hair DNA (Figure 3). In addition to increasing individual detection rates, supplemental video data at hair stations provided information about individual birth and litter year. This increased the opportunity for tracking individual mothers and their offspring over time as cubs transitioned into yearlings and, subsequently, into reproductive‐aged adults (Table 2). Moreover, video data yielded information about cub mortalities that were undetected via hair DNA. This added information increased the accuracy of estimated litter size and offspring apparent survival estimates. Combining maternity information (Figure 3) with capture‐recapture data of offspring (Table 2) also allowed us to evaluate the effects of maternal identity on offspring apparent survival.

### 4.1 Pedigree

Of the 13 adult males that we genetically identified, only five (39%) fathered genetically identified cubs. Costello et al. (2009) similarly found that only 33% of 56 adult male black bears in New Mexico fathered offspring, where older males were more reproductively successful than younger males. We did not know the specific ages of most adult males in our study, but the largest adult male (Bear M2) sired the most offspring, which was consistent with previous findings showing larger male black bears (Kovach & Powell, 2003) and brown bears (Craighead, Paetkau, et al., 1995; Zedrosser et al., 2007) had the highest reproductive success.

In our study, paternity roles appeared to shift through time. During 2012–2014, one adult male (Bear M2) sired all genetically identified offspring (*n* = 6 cubs in 4 litters) and Bears M2 and M5 each fathered three genetically identified cubs born during 2015–2016. In 2018, however, two different adult males (Bears M21 and M15) each fathered three genetically identified cubs and one adult male (Bear M14) sired one genetically identified cub in 2019, whereas Bears M2 and M5 sired none. Beginning in spring 2018, we observed that the largest adult male (Bear M2) sustained a leg injury that caused him to limp throughout 2018. The injury may have made it difficult for Bear M2 to roam widely, an effective strategy for finding and mating with receptive females (Costello et al., 2009; Rogers, 1987; Sandell, 1989), or to compete with males for mating opportunities.

Of the 16 multi‐cub litters, we observed one multiple paternity; Bear M2 sired Bear F17 and Bear M5 sired Bear M23, both offspring were born to Bear F4 in 2017 (Figure 3). Other black bear studies have reported multiple paternities at higher rates than we found; Kovach and Powell (2003) reported multiple paternities in two of seven litters (29%), Costello et al. (2009) found multiple paternities in nine of 32 litters (28%), and Ombrello et al. (2016) reported multiple paternities in three of 15 litters (20%). The relatively low proportion of multiple paternities in our study (6%) may have been biased because we did not genetically identify all littermates in eight of the 16 multi‐cub litters.

Of the 49 individuals for which we identified relationships and included in the pedigree, 22 (45%) did not leave hair DNA. In addition to the expected individual variability in genetic sampling (Khan et al., 2020), we found that one entire age class was almost completely unsampled by hair DNA. Of the 40 offspring first identified via video when they were cubs, only two (5%) rubbed on or otherwise left hair at hair stations when they were ≤1 year old. To a lesser degree, the yearling age class was also relatively unsampled via hair. Thirty‐nine individuals were video‐captured as yearlings during 2010–2020, but only 13 individuals were detected via hair DNA when they were yearlings (Table 3). Almost all offspring that left hair DNA did so when they were 2+ years old. This is important because seven of the eight offspring that died when they were cubs (Figure 3) were never detected via hair DNA. Had we not identified those seven cubs via video, they would have been completely undetected, rendering the pedigree less complete, estimates of offspring apparent survival biased high, and estimates of litter size biased low.

### 4.2 Litter size, interlitter interval, primiparity, natality, and fecundity

We found that mean litter size in our study was 1.95 cubs, comparable to that reported for black bears in Alaska (Miller, 1994), but higher than that reported for other black bear populations in the western US (Baldwin & Bender, 2009; Beecham, 1980; Costello et al., 2003; Jonkel & Cowan, 1971; Kasworm & Thier, 1994). The mean interlitter interval in our study was 2.0 years, similar to most previously reported intervals for black bears in the western US (Baldwin & Bender, 2009; Costello et al., 2003; Hebblewhite et al., 2003; Miller, 1994), but much lower than the 3.2 years reported for black bears in western Montana (Kasworm & Thier, 1994). For their calculation of interlitter interval, Kasworm and Thier (1994) excluded one litter that died, which increased the overall interval mean. When we similarly excluded two litters that died, the mean interlitter interval increased slightly to 2.18 years (95% CI: 1.78, 2.59). The mean age of first reproduction for females in our study was 5 years, the same as that reported for black bears in Alberta (Hebblewhite et al., 2003), but a little lower than that reported for most other black bears in the western USA (Costello et al., 2003; Kasworm & Thier, 1994; Miller, 1994). Mean natality of female bears ≥4 years old was 0.59 cubs/female/year, which was similar to that previously reported for black bears in the Cabinet Mountains and Yaak River of western Montana (0.51; Kasworm & Thier, 1994) and the Whitefish Range of western Montana (0.57; Jonkel & Cowan, 1971), but lower than that reported for black bears in New Mexico (0.78; Costello et al., 2003).

Notably, we combined video data with genetic data from hair, blood, and tissue DNA to determine paternity and confirm maternity for reconstructing the pedigree, but we used only noninvasive hair DNA and video data to estimate reproductive parameters. Therefore, cub mortality that may have occurred in the den was not incorporated into reproductive estimates. If cubs died before we were able to detect them via hair DNA or video data beginning in early spring of each year, then our estimates of mean natality, litter size, and fecundity were likely biased low and our estimates of interlitter interval and age of primiparity were likely biased high.

Our estimate of cub detection rate (via video data) was 0.93, so partial litters were observed during 7% of video‐capture events of family groups. For each family group, the date of the first observation of a partial litter always occurred either after or on the same date we first observed the entire litter together (Table 5). For all multi‐cub litters born after 2012, we video‐captured all littermates together multiple times during year 1. Nonetheless, it was possible that we did not detect all cubs of all litters, which could have affected our reproductive estimates.

Using only noninvasive hair DNA and video data, we estimated fecundity rate, one of the two cornerstones of population biology (Bradshaw & McMahon, 2008). The other cornerstone is survival, which is most informative when partitioned by sex and age classes (Gaillard et al., 2003). We previously used video data cross‐referenced with hair DNA data to estimate the annual apparent survival of black bear yearling males, yearling females, 2+ year‐old males, and 2+ year‐old females in our study site over seven years (Reynolds‐Hogland et al., 2022). Previous bear studies have estimated annual apparent survival, rate of addition or recruitment, and population rate of change using hair DNA and the Pradel model (Boulanger et al., 2002, 2004; McCall et al., 2013; Pederson et al., 2012; Sawaya et al., 2012). We present an alternative approach to estimating reproductive parameters that integrate hair DNA data with video data, which increased our ability to detect the cub and yearling age classes (Table 3). The additional cub information made it possible for us to estimate fecundity rate, which can differ from the rate of addition or recruitment, depending on how many offspring survive and are recruited into the population.

### 4.3 Maternal identity effect on offspring annual apparent survival

Variability in reproduction and survival is an evolutionary adaptation that helps increase population persistence. On our study site, offspring *φ* varied by maternal identity. For example, *φ* of Bear F2’s offspring was significantly lower than *φ* of offspring of most other mothers (Table 6b). Notably, seven of Bear F2’s nine offspring died when they were cubs and none of her female offspring were recruited into the population. In fact, only one of Bear F2’s male offspring (Bear M11) may have survived to pass on genes. Thus, Bear F2’s individual fitness, the expected genetic or phenotypic contribution to future generations (Stearns, 1992), was very low even though she was relatively productive in terms of bearing offspring. Comparatively, Bear F4 also had nine cubs (Figure 3), all of whom transitioned into yearlings. Bear F4 produced six female cubs—Four were born early enough in the study period to determine whether they reached reproductive age. All four survived to reproductive age (≥ 4 years). Of those four, two had 2‐cub litters and all of those cubs survived at least their first year. Bears F1 and F3 also produced female offspring (Figure 3) who subsequently produced offspring that transitioned into yearlings.

The differential *φ* of offspring in our study was not easily explained. Previous bear studies have shown that maternal body mass (Rode et al., 2020) and maternal experience or age (Elowe & Dodge, 1989; Garrison et al., 2007; Johnson et al., 2020; Zedrosser et al., 2009) positively correlated with cub survival. We did not document mass for all adult females in our study because most of our data were noninvasive. However, Bear F2 appeared to be one of the largest adult females that we video‐captured during 2015–2020. In 2020, we live‐trapped Bear F2 and she weighed 77 kg. Also, Bear F2 had a relatively large litter in 2019 (*n* = 3), indicating she was likely in relatively good condition during late fall 2018 (Craighead Sumner et al., 1995; Craighead, Paetkau, et al., 1995; Samson & Huot, 1995). In addition, Bear F2 was nine years old when she lost her three cubs in 2019. Johnson et al. (2020) found that the survival of cubs of middle‐aged black bear mothers was higher compared with the survival of cubs of younger or older mothers, with cub survival highest for offspring of 9‐year‐old mothers. Thus, it seemed unlikely that female body mass, condition, or age accounted for the variability in offspring apparent survival in our study.

Annual fluctuation in food availability can influence bear survival and reproduction (Costello et al., 2003; Eiler et al., 1989; Reynolds‐Hogland et al., 2007). However, it seemed unlikely that annual foods explained the high mortality of Bear F2’s offspring. If annual foods had influenced the apparent survival of Bear F2’s offspring, then we would have expected low apparent survival of the entire cohort, which did not occur.

Differences in spatial use across the landscape by adult females with cubs at heel could also result in differential offspring apparent survival. For example, offspring of mothers that use areas near highways or other high‐traffic roads may experience relatively high mortality due to vehicle strikes. We do not know all the areas that Bear F2 used during 2010–2019. However, we collared Bear F2 during 2020 and collected hourly GPS data. During 2020, Bear F2 did not use areas near highways or other public roads. Bear F2 did use areas near gated gravel roads (within our protected study site), which were rarely used by a few researchers who followed strict protocols to minimize wildlife disturbance.

Maternal care can also influence offspring survival (Balme et al., 2017; Dwyer, 2014; Théoret‐Gosselin et al., 2015), where the length of maternal care is particularly important for bears (Dahle & Swenson, 2003). In our study, maternal care helped explain at least one cub mortality. Video observations of Bear F2 and her single cub in 2016 (Bear F13) revealed that Bear F2 abandoned her seven‐month‐old cub, who died alone in front of a video camera in early August 2016 (Video S1, https://mpgcloud.egnyte.com/dl/DtocMNnaOv). Other studies have reported black bear cub abandonment (Garrison et al., 2007; Lindzey & Meslow, 1980), but our research is the first to document cub abandonment on video.

We documented that Bear F2 abandoned one cub who subsequently died, but Bear F2 may have also abandoned other cubs, given seven of her nine offspring died when they were <1 year old. Thus, we hypothesize that the variability in offspring apparent survival we found may have resulted, in part, from the differential maternal ability to nurture cubs through year one. Previously, Zedrosser et al. (2013) reported no effect of maternal identity on grizzly bear offspring survival. However, Zedrosser et al. (2013) estimated the effect of maternal identity on yearling survival (not cub survival), which they acknowledged may have underestimated the effects of early development on grizzly bear offspring survival.

### 4.4 Integrating video data with genetic data

Integrating video data with genetic data increased the probability of detecting individuals, which helped inform the population‐level pedigree, reproductive parameter estimates, and models of offspring annual apparent survival. Regarding the latter, the estimated *p* based on the top‐ranked model using only genetic data (i.e., null model) was 0.64, which was much lower than the estimated *p* using combined genetic and video data (*p* = .96). Also, the sample size of encounter histories used to model offspring *φ* decreased from 40 (using combined genetic and video data) to 24 (using only genetic data). Moreover, the encounter histories included in the *φ* analysis using only genetic data were truncated for 21 of 24 (88%) offspring. For example, Bear F7 (easily identified because she had a distinct chest blaze) was video‐captured multiple times annually during 2013–2020. Therefore, Bear F7’s annual encounter history (using combined genetic and video data) included nine captures, one for each year, 2013–2020. However, Bear F7 was genetically captured only twice during that same period. Thus, Bear F7’s annual encounter history (using only genetic data) included only two captures. The smaller sample size and truncated encounter histories were likely the reason that the effect of maternal identity on offspring apparent survival was not detected when we used only genetic data. The top‐ranked model from *φ* analyses using only genetic data was the null model, where all other models (including the model that included the effect of maternal identity) ranked very low and had extremely low to zero model weight. Alternatively, the top‐ranked model from *φ* analyses using combined genetic and video data included the effect of maternal identity on offspring apparent survival.

Our study site was relatively small (61 km^2^) and our sampling intensity was relatively high (~2.5 stations/km^2^). Many individuals in our study were captured multiple times at multiple stations annually. Therefore, our sampling intensity may have been excessive for estimating demographic parameters (Reynolds‐Hogland et al., 2022). We do not suggest that other researchers necessarily replicate our high sampling intensity to estimate bear demography. Rather, we suggest that study designs that include hair stations may benefit by adding video cameras at hair stations to increase detection rates (e.g., bears that visit hair stations but do not leave hair can still be detected via video) and provide information on individual age class. We previously provided detailed examples of scaling the use of hair stations supplemented with video cameras for larger study areas (Reynolds‐Hogland et al., 2022). In all examples, the total number of video cameras required to estimate population‐level parameters for large carnivores was well below that considered unrealistic for research programs that use camera data (Gálvez et al., 2016).

### 4.5 Increase in the number of bears detected

During 2013, we video‐captured 20 bears and genetically identified 10 of those bears using hair DNA. By 2020, the number of bears we video‐captured had more than doubled to 42, of which we genetically detected 25. The rapid increase in the number of bears that we video detected and genetically detected on our study site, and the high apparent survival rates, are reasonable for a growing bear population that is protected from hunting and other human disturbance (Reynolds‐Hogland et al., 2022). On our study site, bear harvest had occurred for decades prior to 2009. In 2009, our study site was purchased and immediately transitioned into a conservation property and bear harvest, along with most other human disturbances, were strictly prohibited. Beginning in 2011, sturdy gates were installed on perimeter roads and a security officer patrolled the boundary of our study site. Logging activity in the study area prior to 2009 may also have increased the availability of bear foods, which may also help explain the rapid increase in the number of bears that we detected (Reynolds‐Hogland et al., 2022).

It was not surprising that bear detections via video data were higher than bear detections via hair DNA because not all bears that visit hair stations deposit genetic data. For example, Gurney et al. (2020) reported that 32% of bears that visited hair stations did not leave hair. Also, we used primarily rub trees and rub posts to collect bear hair on our study site. Previous bear rub studies reported that males rubbed on rub trees more than females (Clapham et al., 2012; Rogers, 1987; Seryodkin, 2014; Taylor et al., 2015) and subadult males rubbed on trees less than adult males (Taylor et al., 2015). On our study site, many of the individuals that we video‐detected but did not genetically detect were subadults, yearlings, and cubs.

## CONFLICT OF INTEREST

The authors declare no conflicts of interest.

## AUTHOR CONTRIBUTIONS

**Melissa J. Reynolds‐Hogland:** Conceptualization (equal); Data curation (equal); Formal analysis (lead); Investigation (equal); Methodology (equal); Project administration (equal); Software (lead); Supervision (equal); Validation (lead); Visualization (equal); Writing – original draft (lead); Writing – review & editing (equal). **Alan B. Ramsey:** Conceptualization (equal); Data curation (lead); Formal analysis (equal); Funding acquisition (supporting); Investigation (equal); Methodology (equal); Project administration (lead); Resources (supporting); Software (equal); Supervision (lead); Validation (equal); Visualization (equal); Writing – original draft (supporting); Writing – review & editing (equal). **Carly Muench:** Data curation (equal); Formal analysis (equal); Investigation (equal); Methodology (equal); Writing – original draft (supporting); Writing – review & editing (equal). **Kristine L. Pilgrim:** Conceptualization (equal); Data curation (supporting); Formal analysis (equal); Investigation (equal); Methodology (equal); Project administration (supporting); Software (equal); Supervision (equal); Validation (equal); Writing – original draft (supporting); Writing – review & editing (equal). **Cory Engkjer:** Conceptualization (supporting); Data curation (equal); Formal analysis (equal); Investigation (equal); Methodology (equal); Software (equal); Validation (equal); Writing – original draft (supporting); Writing – review & editing (equal). **Philip W. Ramsey:** Conceptualization (equal); Funding acquisition (lead); Investigation (equal); Project administration (lead); Resources (lead); Supervision (lead); Validation (equal); Visualization (equal); Writing – original draft (supporting); Writing – review & editing (equal).

## Data Availability

All data described herein are available on Dryad (https://doi.org/10.5061/dryad.9p8cz8wjd). Video of American black bear (*Ursus americanus*) cub abandonment in western Montana, USA: Supporting Information (Video S1) available at: https://mpgcloud.egnyte.com/dl/DtocMNnaOv.
